# Corn Responsiveness to *Azospirillum*: Accessing the Effect of Root Exudates on the Bacterial Growth and Its Ability to Fix Nitrogen

**DOI:** 10.3390/plants9070923

**Published:** 2020-07-21

**Authors:** Lucas Caiubi Pereira, Carolina Bertuzzi Pereira, Larissa Vinis Correia, Thaisa Cavalieri Matera, Rayssa Fernanda dos Santos, Cristiane de Carvalho, Elisete Aparecida Fernandes Osipi, Alessandro Lucca Braccini

**Affiliations:** 1 Department of Agronomy, Universidade Estadual de Maringá, Maringá-PR, CEP 87020-900, Brazil; carolbertuzzi14@gmail.com (C.B.P.); pg401097@uem.br (L.V.C.); pg53837@uem.br (T.C.M.); pg53835@uem.br (R.F.d.S.); albraccini@uem.br (A.L.B.); 2 Centro Estadual de Educação Tecnológica Paula Souza, Itú-SP, CEP 13306-220, Brazil; cristiane.carvalho10@etec.sp.gov.br; 3 Center of Agrarian Science, Universidade Estadual do Norte do Paraná, Bandeirantes-PR, CEP 86360-000, Brazil; elisete@uenp.edu.br

**Keywords:** *Azospirillum brasilense*, *Zea mays*, nitrogenase activity, amino acids, sugars, organic acids

## Abstract

Corn has shown different degrees of positive response to inoculation with the nitrogen- fixing bacteria of the genera *Azospirillum*. Part of it has been attributed to the plant genotypic variation, including the root exudates, that are used by the bacteria as energy source. In this study, we grew two corn hybrids that differ for their response to *Azospirillum*, to investigate the effect of different exudates profiles on the bacteria growth and nitrogenase activity. Employing high performance liquid chromatography, we identified nine amino acids (asparagine, aspartic acid, serine, glutamic acid, valine, phenylalanine, threonine, tryptophan and alanine), six sugars (glucose, sucrose, xylose, arabinose, fructose and galactose) and four organic acids (citrate, malate, succinate and fumarate). The less responsive corn genotype showed reduced plant growth (root volume, shoot dry mass and shoot N content), a lower concentration of *Azospirillum* cells within the root tissues, a higher content of asparagine and glucose and a reduced amount of metabolites that serve as bacterial energy source (all organic acids + five sugars, excluding glucose). The genotypes did not interfere in the ability of *Azospirillum* to colonize the substrate, but the metabolites released by the less responsive one reduced the nitrogenase activity.

## 1. Introduction

Symbiosis is a biological phenomenon involving changes in the genome and metabolism of organisms from different species, usually with benefits to one or both [1]. In the plant–microbe relationships, two root symbiotic systems have been actively studied, the arbuscular mycorrhizae and root-associated bacteria [1]. While the first mentioned interaction is probably the most widespread in the ecosystem, the plant association with diazotrophic bacteria has been the more exploited symbiotic relationship in plant production [1,2].

Diazotrophic bacteria comprehend groups of free living, associated and nodule forming species capable of enzymatically reducing the atmospheric nitrogen (N) into plant bioavailable N compounds and, in the agriculture, have been shown capable of enhancing crop yield, while reducing the environmental impacts caused by mineral fertilizers [3,4], such as the production of phytohormones, the N fixation mechanism of these bacteria directly benefit the plant growth; however, some of these bacteria can further provide indirect benefits to plants, including the inhibition of the host pathogenic organisms and the induction of systemic resistance to stresses [5]. Among them, *Azospirillum*, a free living diazotrophic bacterium of the Spirillaceae family, stands out as one of the best characterized and exploited plant-growth promoting microorganism in the agricultural production [3].

To date, twenty species of *Azospirillum* have been described [6], and some have reached commercialization in countries such as Brazil, India and France [3,4]. In the literature, most of the studies on *Azospirillum* focus on the specie *Azospirillum brasilense*, which, since its identification as promising N fixer when associated to grasses crops, has been investigated for its ability to replace nitrogenous fertilizers in sugarcane (*Saccharum* spp.), corn (*Zea mays*), wheat (*Triticum aestivum*), and rice (*Oryza sativa*) [3,6]. However, it is in the corn production that *A. brasilense* stands out as inoculant [6], not only because it allow for a 25% reduction in the need for nitrogenous fertilizer, but also for being capable of increasing the grain yield up to 30% [7]. 

Although largely prospected as N-fixing bacterium [8], nowadays, it is widely accepted that the benefits delivered by *A. brasilense* to cereal plants goes beyond those mechanisms, and includes the mitigation of abiotic stresses, the biological control of plant pathogens, and the production of phytohormones [6]. In the field, the application of *Azospirillum* is either carried out by coating the seeds with a bacterial suspension or by in-furrow soil applications, prior to sowing [9]. Irrespective of the method, a successful inoculation relies on the microorganism’s ability to survive and reproduce in the rhizosphere, a complex environment regulated by the host root-released compounds [10].

Over the cycle, plant roots continuously secrete soluble metabolites, that, based on their molecular weight, are classified into two clusters [11]: one formed by highly diversified group of low-molecular weight metabolites, such as amino acids, organic acids and sugars, and the other constituted of high-molecular weight and less diverse compounds, such as mucilage and proteins [11]. In the genera *Azospirillum*, root exudates play a bimodal role in the rhizosphere colonization, firstly as an energy source, and secondly, serving as chemoattractants that guide the bacterial migration toward the roots [10].

Despite the consistent benefits obtained with the corn inoculation with *Azospirillum*, different degrees of positive outcomes have been reported in the literature [4]. Part of it has been attributed to genotypic variation of the host genetic background, including the composition of the metabolites it releases toward the rhizosphere [12]. Crops genotypes, including corn, may differ in the amount and the composition of their exudates, but the implications of this for the rhizosphere colonization by *Azospirillum*, or for the benefits it provides to plants, remain a poorly-understood subject [13]. 

In this context, hypothesizing that root exudates may be involved in the corn responsiveness to *A. brasilense*, our objective was to investigate the effects of changes in the composition of these metabolites, linked to the crop genotype background on the ability of *Azospirillum* to colonize the rhizosphere, as well as on its N-fixing mechanism.

## 2. Results

We identified nine amino acids (asparagine, aspartic acid, serine, glutamic acid, valine, phenylalanine, threonine, tryptophan and alanine), six sugars (glucose, sucrose, xylose, arabinose, fructose and galactose) and four organic acids (citrate, malate, succinate and fumarate) in the exudates of the tested genotypes. 

The genotypes showed comparable values for total content amino acids (Table 1), but the abundance of each class in the exudates differed with the corn lines (Appendix A). The more responsive one displayed lower content of aspartic acid upon full (100% N), lower alanine concentration under partial (75% N) and higher tryptophan abundance in both the partial N supply and inoculation (75% N + Azo) (Appendix A). In the inoculated plants, comparable results of alanine and threonine were found between the genotypes, while a higher release of asparagine and a lower concentration of aspartic acid, serine, glutamic acid, valine, phenylalanine and tryptophan were identified in the less responsive genotypes (Appendix A).

In relation to sugars and organic acids, the genotypes differed for both, the total content (Table 1) and profile (Appendix B), with the less responsive ones showing a reduced concentration of both and, thus, of chemotactic compounds, i.e., metabolites that serve as chemoattracts and an energy source for *Azospirillum* (sum of all four organic acids + five sugars, excluding glucose). For the less responsive corn line, individual classes of sugar and organic acid were released in lower concentration, apart from glucose, whose concentration was higher upon only mineral N supply (full and partial ) and statistical equivalent to the more responsive one under inoculation (Appendix B).

Regarding plant growth, the full supply of N in the form of mineral fertilizer provided plants showing equivalent results for root volume, shoot dry mass and shoot N content (Table 2). However, upon inoculation, the high responsive genotype showed comparable or higher growth parameters than the full N mineral supply (Table 2), while in the less responsive *Azospirillum*, it was unable to deliver growth parameter, at least, equivalent to mineral fertilization (Table 3). 

Regarding the colonization of the rhizosphere by the bacteria, the genotype background did not play a part in the *Azospirillum* establishment in the substrate, but significantly interfered in the bacteria ability to colonize internal tissues (Table 2), as observed in the reduced most probable number (MPN) of bacteria within the root tissues of the less responsive corn (Table 2). In artificial media (Table 3), the addition of synthetic compounds that mimic the profile of each genotype had no effect on *Azospirillum* count, but interfered in the N-fixing mechanism, with the less responsive genotype showing lower nitrogenase activity (Table 3).

## 3. Discussion

Even though sugars are reported as the main class of root-released compounds by plants [13,14], in the present study, this trend has not been confirmed, as our root exudates contained 60% of amino acids, followed by 30% sugars and 10% organic acids. This contrasting result may be related to the high rates of amino acids released by plant roots at early growth stages, as we sampled the plants at seedling stages [15]. However, this higher amino acid content may also be attributed to the *Azospirillum*, which, as a diazotrophic microorganism, release high rates of N-derivate compounds [16].

Corroborating previous works, all N-depleted plants, as well as those of the less responsive upon inoculation (75% N + Azo), displayed lower amount of sugars, organic acids and chemotactic compounds [17]. All classes of sugars we identified have previously been reported in exudates of corn [18], rice [19] and of the model grass specie *Brachypodium* [20]. Regarding the organic acids, malate, citrate, succinate and fumarate are considered the most common classes released by corn roots [18], and they were all identified in our study. 

In the case of *Azospirillum*, the plant’s root exudates are involved in the bacterial cells migration in the soil [21], in a process governed by energy taxis, i.e., the bacterial moves toward a higher concentration of a nutrient to seek a position at which the energy level is favorable [7,8]. Compared to sugars and organic acids, *Azospirillum* species have a limited reliance on amino acids to grow [4], which, thus, display null or weak attractiveness on the cells [11]. Concerning *A. brasilense*, the species used in this work, all classes of organic acids are considered to exert chemotaxis, while, among the sugars, glucose is not considered a chemoattractant [4] and galactose exerts induced taxis, i.e., only when the cells have previously been grown in media containing it [7,8]. 

However, chemotaxis should not be taken for granted in analyzing corn-*Azospirillum* symbiosis, as some metabolites that do not serve as energy source, or chemoattracts may interfere in the bacterial growth or metabolism, impacting, thus, the benefits delivered by it to plants [4,22,23]. In this context, asparagine that, such as in our work, has been reported as the most abundant amino acid released by corn [18], wheat [24] and the model grass *Brachypodium* [20] roots, has already been reported to impair the activity of the nitrogenase of *Azospirillum* [23]. In cereals, asparagine is involved in N storage and transportation, but its increase within root tissues has been documented as a metabolic response to stress, including nutritional deficiency [25,26].

This is consistent with other plant-growth promoting bacteria, such as *Bacillus*, that had a suppression of bacterial genes involved in the synthesis of protein after changes in the profile of corn exudates [18]. In this work, however, we only investigated the effects of the whole exudates on bacteria growth and nitrogenase activity, but as the less responsive genotype remained, showing a high amount of asparagine, even under inoculation, we suggest that this amino acid may have contributed to reduce the enzyme activity.

Concerning the other amino acids, while aspartic acid have null effects on *Azospirillum* species, the tryptophan benefits the bacterial synthesis of plant growth regulators [23], as this latter compound is structurally and functionally related to the synthesis of auxin, the major phytohormone produced by *A. brasilense* [10,27]. In the literature, an increase in lateral roots due to inoculation has been well-documented and it is, often, ascribed to the bacterial auxin [1,2,5]. In this study, provided the higher concentration of tryptophan in the rhizosphere is of the high responsive genotype, it is plausible that *Azospirillum* may have had been capable of producing higher concentration of auxin [28], compared to the other genotype. This hypothesis is consistent with the results found for plant growth, as the less responsive genotype displayed reduced root volume data upon inoculation. 

Despite the different chemical profiles, the substrate‘s MPN values suggested that any genotype favored *Azospirillum* establishment in the substrate, a trend that was later confirmed by our data on bacterial count in supplemented media. This evidences the great degree of versatility of *Azospirillum* in using different carbon based compounds as energy source, a trait that enables the organism to adapt to a wide array of environments [3], including different agricultural practices and soil types, where cereal crops are grown [4].

*Azospirillum* colonizes all plant parts, but it exhibits a decreasing pattern concentration from roots to leaves [9]. For this reason, the bacteria are generally regarded as a root-surface microorganism, but the species colonizes the roots in a specie-specific way [10]. *A. brasilense*, the species used in this work, can further colonize the interior of root tissues [10], where the nitrogenase is more protected against the irreversible inactivation caused by the oxygen. Supporting Fukami et al. [9], our data on internal root colonization, as estimated by the MPN technique, suggest a genotype-dependency regarding tissues colonization, which, among other factors, depends on the bacterial ability to degrade the structural polysaccharide of cell walls pectin, whose content and structure may differ with the plant genetic background [29]. 

Overall, we showed that just as for their responsiveness, the genotypes differed for the composition of their exudates as well as for the ability of *Azospirillum* to stablish within root tissues. This difference in profile, however, did not affect the ability of *A. brasilense* to stablish in the substrate, or to grow in artificial medium, but it interfered in the nitrogenase activity. In this sense, it is likely that the weak growth performance of the less responsive genotype may be related to its exudates profile (lower relative amount of chemotaxis compounds, lower tryptophan concentration and high relative asparagine content), but also to its lower concentration of *Azospirillum* in the root tissues. 

Our results corroborate that the benefits delivered by *Azospirillum* to corn are affected by the genotype background, and further confirm the involvement of root-released compounds in the degree of response. It is noteworthy that the plant-*Azospirillum* interaction is settled within a dynamic and complex ecosystem constituted by other compounds not identified in this study, but that may interfere in the behavior of microbial communities [14]. Therefore, further researches on the role of root exudates as mediators of the corn-*Azospirillum* symbiosis need to be carried out, including regarding the amino acids classes, as despite their null chemotaxis, they seemed to be determinant in the degree of the benefits provided by *A. brasilense.* Additionally, rather than being the cause of the responsiveness, root exudates are more likely to reflect the specificity of transcriptional signaling involved in the mutual recognitions between *Azospirillum* and its host plant [12,13].

## 4. Materials and Methods

We tested two inbred lines showing different N use efficiency under *Azospirillum* inoculation, as described by Zeffa et al. [30] and Vidotti [13]. The lines belong to the germplasm bank of the *Universidade Estadual de Maringá* (Brazil), and were obtained from successive self-pollinations of commercial hybrids with tropical background. Based on the work reported by Zeffa et al. [30], we selected the line L7 as a high responsiveness hybrid to inoculation, while L16 was taken as the low one.

Seeds of both hybrids were sown in Leonard jars [31] containing sterilized substrate (sand and pulverized coal at 3:1 v/v) and sterile nutrient solution. Following Zeffa et al. [30], the nutrient solution contained (L^−1^): 2.0 mmol Ca(NO_3_)_2_ (calcium nitrate tetrahydrate, Sigma-Aldrich, Saint-Louis, MO, USA); 0.75 mmol K_2_SO_4_ (potassium sulfate, Merck Millipore, Darmstadt, Germany); 0.65 mmol MgSO_4_ (magnesium sulfate heptahydrate, Sigma-Aldrich); 0.1 mmol KCl (potassium chloride BioXtra, Sigma-Aldrich); 0.25 mmol KH_2_PO_4_ (potassium phosphate monobasic, Supelco, Darmstadt, Germany); 1 × 10^−3^ mmol H_3_BO_3_ (boric acid BioXtra, Sigma-Aldrich); 1 × 10^−3^ mmol MnSO_4_ (sulfate monohydrate BioReagent, Sigma-Aldrich); 1 × 10^−4^ mmol CuSO_4_ (cupric sulfate pentahydrate, Sigma-Aldrich); 1 × 10^−3^ mmol ZnSO_4_ (zinc Sulfate, J.T.Baker, Center Valley, PA, USA); 5 × 10^−6^ mmol (NH_4_)_6_Mo_7_O_24_ (ammonium molybdate tetrahydrateand, Sigma-Aldrich) and 0.1 mmol Fe-EDTA (Fe–EDTA, BioReagent, Sigma-Aldrich).

As bacterial source, a liquid formulation of *Azospirillum brasilense* containing a blend of the AbV5 and AbV6 strains was purchased at the local market (Masterfix Gramíneas, Stoller do Brasil, Campinas, Brazil). These strains derive from a Brazilian nationwide selection program for corn, and allow for a replacement of 25% of the total input of the needed nitrogenous fertilizer [3]. The crop inoculation was manually performed by mixing 100 mL of the commercial product with 1 kg of corn seeds in order to obtain 1.2 × 10^5^ of viable cells seed^−1^. We tested three N management conditions: (i) full solution, i.e., supplying 2.0 mmol L^−1^ Ca (NO_3_)_2_ (100% N); (ii) partial N supply, i.e., only 75% of Ca(NO_3_)_2_ used in the full solution (75% N) and (iii) 75% of N + *Azospirillum brasilense* inoculation (75% N + Azo). Under natural light and temperature conditions, the plants were grown in a greenhouse, where the recorded daily mean temperature ranged between 25 °C and 31 °C, and the daily mean air relative humidity ranged between 62% and 78%. After 25 days, we performed the following evaluations: root volume, shoot dry mass, shoot N content, rhizosphere bacterial count (soil and root tissues) and root exudate profiling.

### 4.1. Plant Growth Parameters

The root volume was determined as the difference between the water volume within a graduated cylinder before and after insertion of the two-times washed fresh roots [30]. The shoot parts were stored in paper bags and dried in a forced ventilation oven at 60 °C for 72 h for subsequent determination of the dry mass and its N content by the Kjeldahl digestion method [32]. Next, after weighting, the dried shoot was ground in a mill for 60 s at 17,000 rpm; then 0.2 g of the flour was placed in test tubes containing 2 g of a catalysis (copper sulfate and selenium powder) and 5 mL of concentrated sulfuric acid. Then, the tubes were gradually heated up to 350 °C on a block digestor for 2.5 h, up to the digestion phase for organic matter. Afterwards, the released ammonia distillation phase was started by a reaction to sodium hydroxide (50%), which was then collected in 4% boric acid solution. Finally, titration was carried out in standard chlorohydric acid solution (1 mol L^−1^) and the difference of the amount of nitrogen was calculated.

### 4.2. Rhizosphere Colonization

For bacterial count we used the most probable number technique (MPN) described by Dobereiner et al. [33]. To evaluate the substrate colonization, 10 g of the root surrounded substrate were collected and suspended in 45 mL saline solution (NaCl 0.85%), while, for assessing the internal the colonization of internal tissues, roots tips were collected and five times washed with sterile distilled water and surface-disinfected by immersion in 70% ethanol (30 s), followed by an 8-min gentle agitation in 2% sodium hypochlorite. Then, 10 g of this root tissue were macerated and suspended in 45 mL saline solution (NaCl 0.85%).

For both the substrate and the root tissues, the saline solution was submitted to serial dilutions from 10^−1^ to 10^−8^. Then, 0.1 mL aliquot of each suspension was inoculated onto a semisolid nitrogen-free medium [33], which was incubated in the dark at 30 °C for 72 h. After that period, the bacteria concentration was estimated, based on the biofilm formation employing the McCrady probability table [33]. We used the solid culture media NFb that favors the growth of the used strains as well as of other *Azospirillum* species [33], and its composition was as follow (L^−1^): malic acid 5.00 g (D-malic acid, ReagentPlus, Sigma-Aldrich, Saint-Louis, MO, USA), KOH 4.00 g (potassium hydroxide BioXtra Sigma-Aldrich), K_2_HPO_4_ 0.50 g (potassium phosphate dibasic, Sigma-Aldrich), FeSO_4_·7H_2_O 0.05 g (iron(II) sulfate heptahydrate, ReagentPlus-Sigma-Aldrich), MnSO_4_·7H_2_O 0.01g (magnesium sulfate heptahydrate, ReagentPlus, Sigma-Aldrich), MgSO_4_·7H_2_O 0.10g (magnesium sulfate heptahydrate, ReagentPlus, Sigma-Aldrich), NaCl 0.02 g (sodium chloride, ACS, Sigma-Aldrich), CaCl_2_ 0.01 g (calcium chloride, Sigma-Aldrich), Na_2_MoO_4_ 0.002 g (sodium molybdate, Sigma-Aldrich), bromothymol blue 0.5% in 95% methanol 2.00 mL (bromothymol blue, ACS reagent-Sigma-Aldrich), agar 1.8 g (agar powder, Sigma-Aldrich) and NH_4_Cl 0.7 g (ammonium chloride, Sigma-Aldrich).

### 4.3. Exudates Collection and Analyses

The exudates were collected by immersing the whole root system in 100 mL of sterile ultra-pure water (Water Ultrapur, Supelco, Darmstadt, Germany), where it remained for 3 h, under stirring in an orbital shaker (60 rpm). Then, following Kawasaki et al. [20], the liquid solution was filtered (PHENEX RC syringe filter, Allcrom, São Paulo, Brazil) and submitted high performance liquid chromatography (HPLC) to quantify the amino acids, the sugars and the organic acids contents released by each genotype. The HPLC was performed as follow: HP 1100, chromatographic column: 4.0 × 125 mm C18, temperature of column: 40 °C, velocity of flow: 1.0 mL min^−1^, wavelength: 338 nm, 262 nm (Pro), mobile phase A: 20 mmol sodium acetate solution (sodium acetate for HPLC, LiChropur, Darmstadt, Germany) phase B: 20 mmol (1:2:2 (v/v/v)) of sodium acetate solution: methanol (methanol for HPL, LiChropur): acetonitrile (acetonitrile for HPC, LiChropur).

### 4.4. Nitrogenase Activity

To test the effects of each genotype root-released on the *Azospirillum* growth and the nitrogenase activity, 1 mL of the neat inoculant was inoculated on semisolid NFb medium, supplemented with compounds mimicking the exudates of each genotype under N deprivation (75%). The concentration of each compound used in the media preparation (nmol L^−1^) was based on their abundance, according to the HPLC results (Appendixes A and B). Neat NFb medium was used as control and, as amino acids source we used L-asparagine (BioReagent, Sigma-Aldrich), L-aspartic acid (BioReagent, Sigma-Aldrich), L-serine (Sigma-Aldrich), L-glutamic acid (ReagentPlus, Sigma-Aldrich), D-valine (BioReagent, Sigma-Aldrich), L-phenylalanine (non-animal source, Sigma-Aldrich, L-threonine (PharmaGrade, SAFC); L-tryptophan (non-animal source, Sigma-Aldrich) and L-Alanine (Sigma-Aldrich). As a source of sugars, we used D-glucose (Sigma-Aldrich), sucrose (BioReagent, Sigma-Aldrich), arabinose (Pharmaceutical Secondary Standard, Supelco), xylose (European Pharmacopoeia Reference Standard, Sigma-Aldrich), D-fructose (BioReagent, Sigma-Aldrich) and D-dalactose (BioReagent, Sigma-Aldrich). The organic acids (citrate, malate, succinate and fumarate) were purchased from Sigma-Aldrich, BioReagent line.

In accordance with Kim et al. [34], we used the acetylene-reduction assay to assess the nitrogenase activity, which is an indirect method that uses the enzyme’s ability to reduce acetylene gas to ethylene. Then, we incubated the NFb media for 48 h at 30 °C in the dark to then reduce their gas phase’s partial pressure of oxygen with a mixture of acetylene-air-N (10:10:80 v/v/v) (Bovine Serum Albumin, Sigma-Aldrich). Finally, after 24 h, the rate of ethylene production using gas chromatograph with a flame ionization detector HP-PLOT/AL203 column, and the MPN of *Azospirillum* were measured.

### 4.5. Experimental Design and Statistical Analyses

In the greenhouse, the experiment was conducted in the completely randomized design in a 2 × 3 factorial scheme, with four replications: two genotypes of different responsiveness × three N managements. From individual HPLC results we further calculated the content of chemotactic compounds, i.e., the concentration of metabolites that serve as bacterial energy source (all organic acids + five six sugars, excluding glucose). When investigating the impact of genotype exudates on *Azospirillum* growth and N fixation, we tested three treatments in the completely randomized design: one neat NFb medium as a control and two supplemented NFb media, each, containing compounds representing the exudates upon 75% N. After checking the normality and homogeneity of variances, the means were compared using the Tukey test at a 5% probability level. To individually compare the content of each class of metabolites in the exudates, the F-test was conclusive.

## Figures and Tables

**Table 1:** Means of total amino acids content (TAA), total sugar (TS), total organic acids (TOA), total of chemotaxis compounds (TCC), most probable number of diazotrophic bacteria (MPN), root volume (RV), shoot dry mass (SDM) and its N content (NCSDN) of two corn genotypes of high (HResp) and low (LResp) responsiveness to *A. brasilense* under full N mineral supply (100% N), N deprivation (75% N) as well as partial N supply coupled with inoculation (75% N + Azo).

Treatments	TAA (nmol g^−1^)	TS (nmol g^−1^)		TOA (nmol g^−1^)		TCC * (nmol g^−1^)	
	HResp + LResp	HResp	LResp	HResp	LResp	HResp	LResp
100% N	1633.15C	1062.3Ba	952.82Ab	394.88Ba	216.07Ab	686.13Ba	310.8Ab
75% N	1040.15B	776.52Ca	750.60Ba	212.85Ca	116.68Bb	339.07Ca	170.18Bb
75% N + Azo	1770.18A	1125.98Aa	928.2Ab	447.42Aa	209.95Ab	724.11Aa	298.8 Ab
Means	1481.16	988.27	877.21	351.72	180.90	583.10	259.93
CV (%)	2.67	2.00		3.25		2.19	

According to the Tukey test, means followed by the same capital letter in the column belong to the same group at 5% probability. Means followed by the same lowercase letters in the row do not differ from each other by the F-test at a 5% probability level. * Sum of sugars (excluding glucose) + organic acids.

**Table 2:** Means of root volume (RV), shoot dry mass (SDM), shoot N content (NCSDN) and of the most probable number of *Azospirillum* (MPN) in the substrate and root tissue of two corn genotypes of high (HResp) and low (LResp) responsiveness to *A. brasilense* under full N mineral supply (100% N), N deprivation (75% N), as well as partial N supply coupled with inoculation (75% N + Azo).

Treatments	RV (mL)		SDM (g)		NCSDM (mg)		MPN Substrate	MPN Root Tissue	
	HResp	LResp	HResp	LResp	HResp	LResp	HResp + LResp	HResp	LResp
100% N	22.34Ba	22.56Aa	9.31Ba	9.34Aa	10.54Aa	10.34Aa	0.31B	0.01Ba	0.01Ba
75% N	10.54Ca	11.01cb	7.34Ca	7.45Ca	7.34Ba	6.65Cb	0.36B	0.01Ba	0.01Ba
75% N + Azo	26.87Aa	16.89Bb	11.34Aa	8.56Bb	10.91Aa	7.56Bb	8.45Aa	1.12Aa	0.45Ab
Means	19.58	18.82	9.33	8.45	9.60	7.85	3.03	0.28	
CV (%)	10.23		8.95		4.34		5.45	8.45	

According to the Tukey test, means followed by the same capital letter in the column belong to the same group at 5% probability. Means followed by the same lowercase letters in the row do not differ from each other by the F-test at a 5% probability level.

**Table 3:** Most probable number of *Azospirillum* (MPN) and nitrogenase activity of *A. brasilense* grown in neat NFb medium and NFb media supplemented with synthetic compounds mimicking the more responsive genotype (NFb + HResp) as well as the less responsive one (NFb + LResp).

Growth Media	MPN (10^8^ Cells g^−1^)	Nitrogenase Activity (nmol C_2_H_4_·mg^−1^ h^−1^)
Neat NFb	18.56A	387.21A
NFb + HResp	20.54A	365.34A
NFb + LResp	19.56A	267.23B
Mean	19.55	339.93
CV (%)	9.34	12.45

According to the Tukey test, means followed by the same capital letter in the column belong to the same group at 5% probability.

## Appendix A

**Table A1:** Means of asparagine, aspartic acid, serine, glutamic acid, valine, phenylalanine, threonine, tryptophan and alanine of two corn genotypes of high (HResp) and low (LResp) responsiveness to *A. brasilense* under full N mineral supply (100% N), partial N (75% N) as well as partial N supply coupled with inoculation (75% N + Azo).

Amino Acids (nmol g^−1^)					
Classes	N Management	HResp	LResp	Means	CV (%)
Asparagine	100% N	667.55a	687.75a	677.65	5.34
	75% N	897.15a	886.10a	891.63	6.35
	75% N + Azo	877.68b	1286.05a	1081.86	8.82
Aspartic Acid	100% N	487.43b	557.00a	522.21	7.34
	75% N	115.48a	114.90a	115.19	8.36
	75% N + Azo	428.93a	290.75b	359.84	8.23
Serine	100% N	149.50a	140.18a	144.84	11.34
	75% N	10.30a	10.35a	10.32	10.94
	75% N + Azo	133.08a	83.05b	108.06	8.34
Glutamic acid	100% N	155.75a	163.85a	159.80	6.74
	75% N	8.80a	9.08a	8.94	8.56
	75% N + Azo	142.60a	85.55b	114.08	9.31
Valine	100% N	47.20a	45.53a	46.36	6.45
	75% N	5.98a	5.70a	5.84	7.01
	75% N + Azo	55.43a	30.40b	42.91	5.64
Phenylalanine	100% N	34.80a	34.83a	34.81	5.56
	75% N	4.05a	4.33a	4.19	6.31
	75% N + Azo	32.75a	19.48b	26.11	6.54
Threonine	100% N	27.90a	25.78a	26.84	5.64
	75% N	3.00a	2.93a	2.96	5.67
	75% N + Azo	24.98a	13.75a	19.36	9.34
Tryptophan	100% N	11.63a	12.45a	12.04	8.34
	75% N	0.93a	0.60b	0.76	6.45
	75% N + Azo	11.80a	9.43b	10.61	7.46
Alanine	100% N	8.35a	8.85a	8.60	8.74
	75% N	0.20b	0.45a	0.33	9.56
	75% N + Azo	7.25a	7.43a	7.34	7.45

According to the F-test, means followed by the same letter in the row do not differ from each other at a 5% probability level.

## Appendix B

**Table A2:** Means of sugars (glucose, sucrose, xylose, arabinose, fructose and galactose) and organic acids (citrate, malate, succinate and fumarate) of two corn genotypes of high (HResp) and low (LResp) responsiveness to *A. brasilense* under full N mineral supply (100% N), partial N (75% N) as well as partial N supply coupled with inoculation (75% N + Azo).

N Management	Sugars (nmol g^−1^)					Organic Acids (nmol g^−1^)				
		HResp	LResp	Means	CV (%)		HResp	LResp	Means	CV (%)
100% N	Glucose	771.05b	858.10a	814.58	6.34	Citrate	198.58a	107.60b	153.09	6.67
75% N		650.30b	697.10a	673.70	6.56		129.23a	83.90b	106.56	6.34
75% N + Azo		849.30a	839.35a	844.33	7.34		206.03a	102.68b	154.35	5.34
100% N	Sucrose	106.38a	51.20b	78.79	5.45	Malate	140.15a	72.90b	106.53	6.76
75% N		82.23a	31.73b	56.98	6.35		70.88a	21.60b	46.24	7.96
75% N + Azo		109.15a	43.23b	76.19	5.56		175.35a	65.00b	120.18	9.45
100% N	Arabinose	78.05a	23.25b	50.65	6.65	Succinate	45.91a	30.33b	38.12	5.68
75% N		23.28a	8.93b	16.10	5.45		11.00a	10.41b	10.71	7.41
75% N + Azo		75.25a	23.30b	49.28	6.12		54.48a	31.25b	42.86	5.01
100% N	Xylose	47.25a	10.78b	29.01	4.54	Fumarate	10.25a	5.25b	7.75	4.46
75% N		11.63a	5.13b	8.38	5.34		1.75a	0.78b	1.26	4.89
75% N + Azo		44.40a	12.55b	28.48	5.67		11.58a	11.03b	11.30	5.46
100% N	Fructose	54.73a	8.90b	31.81	6.87					
75% N		7.80a	7.50b	7.65	4.78					
75% N + Azo		44.31a	9.25b	26.78	7.45					
100% N	Galactose	4.85a	0.60b	2.73	6.45					
75% N		1.30a	0.23b	0.76	7.56					
75% N + Azo		3.58a	0.53b	2.05	7.56					

According to the F-test, means followed by the same letter in the row do not differ from each other at a 5% probability level.
